# Annual Meetings of Hospitals

**Published:** 1921-03-12

**Authors:** 


### Annual Meetings of Hospitals.

### The Central London Ophthalmic Hospital.

The seventy-seventh annual report of the Central London Ophthalmic Hospital, presented at a meeting presided over last week by Mr. C. Murray Wilkins, states that out-patients during the period under review numbered 13,315, and the total attendances was 30,715, as against 13,008 patients, with attendances totalling 29,383, in 1919. In-patients numbered 594, and were 106 in excess of those admitted in 1919. A new *x*-ray apparatus has been installed, together with a localiser and all necessary adjuncts. During the past year twenty-seven students availed themselves of the classes and lectures which have been arranged in conformity with the requirements of the conjoint Board of the Royal College of Physicians and Surgeons for the diploma in ophthalmic medicine and surgery and with the University of London for the degree of M.S. in ophthalmology. An earnest appeal was made for increased financial support. To carry on the work efficiently an annual sum of at least £5,500 is required.

### St. Mary's Hospital for Women and Children, Plaistow.

The Bishop of Barking presided at the annual meeting of St. Mary's Hospital for Women and Children, Plaistow. The accounts show a deficit of £1,205, the expenditure for the year having amounted to £8,748, whereas the income was £7,543 only. That deficit, together with the losses of previous years, was met by the sale of General Fund Investments. In-patients admitted during the year under review totalled 787, as against 716 in the previous year. The weekly cost per bed for in-patients amounted to £2 16s. 8d., against £1 9s. 4d. in 1914. An urgent appeal was made for increased financial support to enable the projected nurses' home and out-patients' department to be carried out. The Earl of Macclesfield has accepted the office of Hon. Treasurer, and Mr. John Weir will be the new Chairman of the Committee of Management.

### King's College Hospital.

Lord Hambleden, presiding at the annual meeting of King's College Hospital, referred to the serious financial position of the institution. Special grants from the King's Fund and the National Relief Fund had reduced the deficit to £19,365. The report, which was adopted, stated that donations, including the Camberwell War Memorial grant of £2,000, amounted to £8,000 as compared with £4,000 in 1919. Payments by patients totalled £186 per week, and would, it was estimated, reach £300 a week in 1921. Although the cost of living was officially given as 176 per cent. above the pre-war standard, cost of provisions per patient, which in 1914 was 12s. 6d. per week, amounted in 1920 to only 18s. 6d. Interest on a loan of £160,000 formed an annual charge on the hospital of £7,000. In-patients in 1920 totalled 6,675, as compared with 4,955 in 1919. Out-patient attendances were 196,200, as compared with 145,170 during the previous year. Special clinics had been reserved for ex-Service men, who much appreciated that arrangement. The ante-natal department, the radiological department, the special department for the treatment of children's diseases, and the venereal disease department were all doing useful and increasing work. The hospital has formed an Old Patients' League; all patients before leaving the institution are asked to enrol their names, pay a minimum subscription of 5s. per annum, and undertake to endeavour to persuade three friends to join.

